# Sex Differences in Autonomic Cardiac Control and Oxygen Saturation Response to Short-Term Normobaric Hypoxia and Following Recovery: Effect of Aerobic Fitness

**DOI:** 10.3389/fendo.2018.00697

**Published:** 2018-11-23

**Authors:** Michal Botek, Jakub Krejčí, Andrew McKune

**Affiliations:** ^1^Department of Natural Sciences in Kinanthropology, Faculty of Physical Culture, Palacký University Olomouc, Olomouc, Czechia; ^2^Discipline of Sport and Exercise Science, School of Rehabilitation and Exercise Sciences, Research Institute for Sport and Exercise Science, University of Canberra, Canberra, ACT, Australia; ^3^Discipline of Biokinetics, Exercise and Leisure Sciences, School of Health Sciences, University of KwaZulu-Natal, Durban, South Africa

**Keywords:** gender, autonomic nervous system, vagal activity, sympathovagal balance, simulated altitude, heart rate variability, maximal oxygen uptake

## Abstract

**Introduction:** The main aims of this study were to investigate autonomic nervous system (ANS) and arterial oxygen saturation (SpO_2_) responses to simulated altitude in males and females, and to determine the association between maximal oxygen uptake (VO_2_max) and these responses.

**Materials and Methods:** Heart rate variability (HRV) and SpO_2_ were monitored in a resting supine position during Preliminary (6 min normoxia), Hypoxia (10 min, fraction of inspired oxygen (FiO_2_) of 9.6%, simulated altitude ~6,200 m) and Recovery (6 min normoxia) phases in 28 males (age 23.7 ± 1.7 years, normoxic VO_2_max 59.0 ± 7.8 ml.kg^−1^.min^−1^, body mass index (BMI) 24.2 ± 2.1 kg.m^−2^) and 30 females (age 23.8 ± 1.8 years, VO_2_max 45.1 ± 8.7 ml.kg^−1^.min^−1^, BMI 21.8 ± 3.0 kg.m^−2^). Spectral analysis of HRV quantified the ANS activity by means of low frequency (LF, 0.05–0.15 Hz) and high frequency (HF, 0.15–0.50 Hz) power, transformed by natural logarithm (Ln). Time domain analysis incorporated the square root of the mean of the squares of the successive differences (rMSSD).

**Results:** There were no significant differences in SpO_2_ level during hypoxia between the males (71.9 ± 7.5%) and females (70.8 ± 7.1%). Vagally-related HRV variables (Ln HF and Ln rMSSD) exhibited no significant differences between sexes across each phase. However, while the sexes demonstrated similar Ln LF/HF values during the Preliminary phase, the males (0.5 ± 1.3) had a relatively higher (*p* = 0.001) sympathetic activity compared to females (−0.6 ± 1.4) during the Hypoxia phase. Oxygen desaturation during resting hypoxia was significantly correlated with VO_2_max in males (*r* = −0.45, *p* = 0.017) but not in females (*r* = 0.01, *p* = 0.952) and difference between regression lines were significant (*p* = 0.024).

**Conclusions:** Despite similar oxygen desaturation levels, males exhibited a relatively higher sympathetic responses to hypoxia exposure compared with females. In addition, the SpO_2_ response to resting hypoxia exposure was related to maximal aerobic capacity in males but not females.

## Introduction

Sojourning at high altitude is a popular pursuit for many people around the world, either as part of a vacation (mountain climbing, skiing, trekking) or as a popular strategy for improving aerobic performance, using various altitude training approaches, in elite and/or amateur endurance athletes (1–3). Due to the improvement in transportation, an increasing number of people can travel passively to high altitude for short visits, for instance by lift, car, and/or helicopter without previous training and/or acclimatization, and this may be a risk for acute mountain sickness (AMS) development (4). During the sojourn to high altitude, it is important to consider that a low oxygen environment represents an added stress to the body (5), and tolerability to high altitude exposure in human beings seems is highly variable (6–10). Both lower atmospheric pressure or low fraction of inspired oxygen (FiO_2_) induces a progressive decline in arterial oxygen saturation (SpO_2_), causing immediate compensatory responses in the pulmonary and cardiorespiratory systems to ensure the adequate supply of oxygen to vital tissues (11). An acute hypoxia ventilatory response (AHVR) is thought to be a vital body response for homeostatic SpO_2_ adjustment during hypoxia (12). It was demonstrated that AHVR is augmented by hypercapnia (13), and in the population, AHVR is characterized with great inter-individual variability in hypoxic chemosensitivity (14). Some authors have associated a progressive decline in SpO_2_ with AMS at high altitude (15), while others associate AMS with sympathetic dominance in autonomic cardiac control (5). Previously, a higher AMS incidence was positively associated with higher VO_2_max level in mountain climbers (15). In this regard, it has been established that the SpO_2_ response to hypoxia during exercise is negatively affected by higher aerobic capacity in both males (8, 16, 17) and females (18). This relationship is commonly explained as a result of the relative hypoventilation mediated by blunted chemoreceptor sensitivity in individuals with higher VO_2_max (17, 19, 20). However, there is conflicting evidence as to sex differences relating to the role of aerobic capacity in moderating the SpO_2_ response during resting normobaric hypoxia exposure (17, 18). The acute homeostatic adjustment to systemic hypoxia is a complex stress-regulated response, which is primarily mediated by a central command mechanism (21) and changes in autonomic nervous system (ANS function) (12). The functional changes in the ANS at altitude are considered to be an adaptive response to hypoxia (22, 23) as well as a response to hypoxia inducible factor 1α production at the cellular level (24). These changes modulate metabolic pathways as well as immune responses that play an important part in adaptation response to hypoxia (25).

Spectral analysis of R-R interval to determine heart rate variability (HRV) is commonly accepted as a non-invasive tool for autonomic cardiac control assessment (26), especially parasympathetic (vagal) cardiac outflow (27). Vagal activity is reflected in both high-frequency power (HF, 0.15–0.50 Hz) of R-R intervals and/or in time domain root mean square of the successive R-R interval differences (rMSSD), and is associated with respiratory modulated fluctuation of heart rate (HR) that causes a phenomenon known as respiratory sinus arrhythmia (RSA) (28). Low frequency power (LF, 0.05–0.15 Hz) is considered to be modulated by baroreflex activity (29) together with bilateral sympathetic and vagal traffic (30). Ratio LF/HF is traditionally thought to be an index of sympathovagal balance (31, 32). The hypoxia-induced increase in resting HR seems to be a result from a decrease in cardiac vagal activity and an increase in relative sympathetic activity (10, 33–35). From a medical standpoint, it is well-known that long-term sympathetic predominance in autonomic cardiac regulation contributes to increasing risk of cardiovascular disease such as malignant arrhythmias (36), hypertension (37), and/or sudden cardiac death (38). A recently published meta-analysis demonstrated that healthy females showed a higher resting HR accompanied with lower global autonomic activity compared with age matched males. However, at rest, females maintained a significantly greater HF and lower LF power, that was further reflected by a lower LF/HF ratio, in normoxia conditions, representing a cardio-protective effect of vagal activity (39). Regarding, hypoxia-induced gender differences in HRV, to date, published data is inconsistent. For example Wadhwa et al. (40) demonstrated a visible decrease in vagal activity, with a concomitant increase in sympathetic cardiac control, in males compared with females, in response to intermittent normobaric hypoxia exposure (FiO_2_ = 8.0%) and also during the recovery period. However, more recently, Boos et al. (41) found higher overall autonomic cardiac activity during ascent to high terrestrial altitude in males compared with age, body mass index (BMI) matched females. In addition, females and males demonstrated a similar cardiopulmonary responses during 150 min of normobaric hypoxia exposure (FiO_2_ = 11.5%) (42).

Therefore, our primary objective was to test the hypothesis that age-matched females and males exhibit no differences in autonomic cardiac and SpO_2_ response to equal simulated altitude. In addition, our secondary objective was to test the hypothesis that there are no sex differences in SpO_2_ response to resting hypoxia in relation to VO_2_max level.

## Materials and methods

### Participants

The study included 28 males and 30 females. Data for males were published previously (9) and reanalysed for comparison with females for the purpose of this study. Somatic and physiological characteristics of the experimental group are presented in Table 1. Subjects were healthy non-smoking, sport science students, who had not been exposed to hypoxia for at least the previous 2 years and were not on any medication or dietary supplements. They underwent preliminary medical screening to identify cardiovascular, pulmonary, and metabolic conditions that would exclude them from the study. This study was carried out in accordance with the recommendations of Ethics Committee of the Faculty of Physical Culture, Palacký University Olomouc. The protocol was approved by the Ethics Committee of the Faculty of Physical Culture, Palacký University Olomouc. All subjects gave written informed consent in accordance with the Declaration of Helsinki.

**Table 1:** Anthropological and physiological characteristics of studied groups.

	**Females**	**Males**	***p*-value**	**ES**	**ES rating**
*n*	30	28		
Age (years)	23.8 ± 1.8	23.7 ± 1.7	0.812	0.06	Trivial
Weight (kg)	60.8 ± 8.5	78.4 ± 7.9	<0.001	−2.16	Large
Height (cm)	167.0 ± 5.6	180.3 ± 7.2	<0.001	−2.07	Large
BMI (kg.m^−2^)	21.8 ± 3.0	24.2 ± 2.1	0.001	−0.90	Large
Fat (%)	20.5 ± 6.2	12.6 ± 4.8	<0.001	1.41	Large
FFM (%)	79.5 ± 6.2	87.4 ± 4.8	<0.001	−1.44	Large
VO_2_max (ml.kg^−1^.min^−1^)	45.1 ± 8.7	59.0 ± 7.8	<0.001	−1.67	Large
HRmax (beats.min^−1^)	191.3 ± 8.1	190.0 ± 6.9	0.490	0.18	Trivial
VC (l)	4.35 ± 0.64	6.18 ± 0.70	<0.001	−2.74	Large

Values are presented as mean ± SD.

*ES, effect size (Cohen's d); n, sample size; BMI, body mass index; FFM, fat free mass; VO_2_max, maximal oxygen uptake; HRmax, maximal heart rate; VC, vital capacity*.

### Experimental protocol

The subjects were required to avoid eating, drinking coffee, tea, and/or any substance affecting the ANS activity for at least 2 h before the experiment. In addition, they were asked to avoid vigorous physical activity and alcohol for 48 h before the experiment. The experiment was performed between 8:00 and 11:00 a.m. in a laboratory where the ambient temperature ranged from 22 to 24°C. During the experiment, each subject rested quietly in a supine position and was shielded against acoustic and visual disturbances.

From a methodological perspective, the influence of breathing patterns and tidal volume on the HRV components is well-described (43, 44) where a decrease in breathing frequency (BF) and increased tidal volume cause an increase in the HF component. However, a BF <9 breaths per minute may lead to an artificial increase in the LF with concomitant changes in the LF/HF ratio due to an RSA peak shift from HF into the LF. This could be a limitation in terms of interpreting both the vagal and the sympathetic contribution to the sinoatrial node activity (45). To avoid the potential methodological issue of BF, HRV is frequently measured under paced breathing conditions (7, 46). However, it has been reported that paced breathing may increase sympathetic activity (47). Therefore, in the present study we used spontaneous breathing throughout the experimental protocol. In respect to methodological issues mentioned above, 4 males and 3 females were excluded from the original dataset of 32 males and 33 females due to BF <9 breaths.min^−1^ within any phase of the experiment.

In order to minimalize a potential effect of the menstrual cycle to HRV results (48), all females were measured similarly at the follicular phase of the menstrual cycle based on self-report. In addition, 6 (20%) participating females were taking oral contraceptives pills.

**Figure 1:**
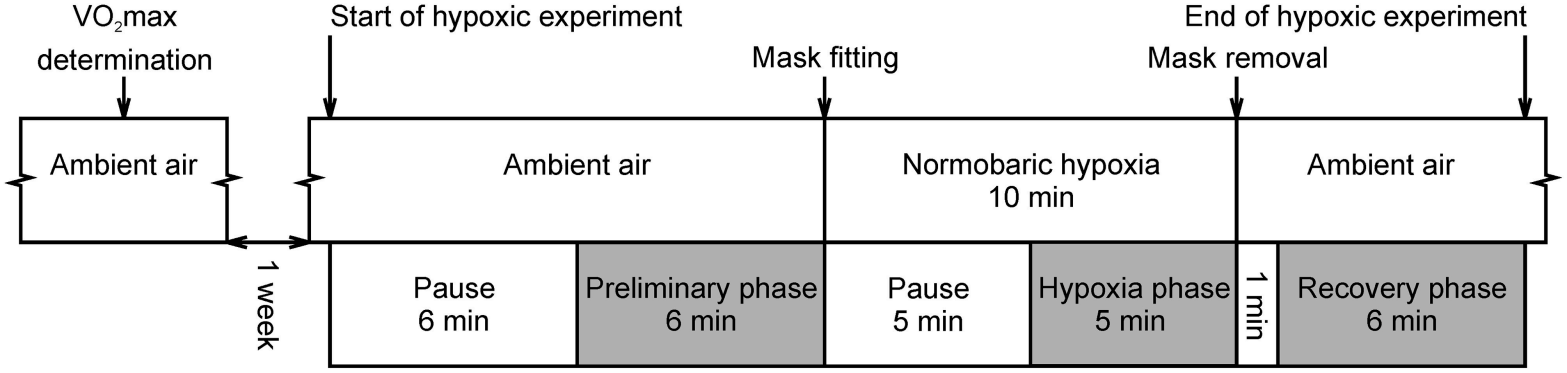
Course of experimental protocol. Gray colored phases were intended for oxygen saturation and ECG recording. Pauses were used to skip the transitory phase and to be able to assume stationarity of the data. This is a reprint of the figure entitled “Course of hypoxic experimental protocol” by Macoun et al. (9) and licensed under CC BY 4.0 (http://creativecommons.org/licenses/by/4.0/).

The hypoxic experiment proceeded as follows (Figure 1): The subjects first breathed ambient air without a breathing mask. Each subject lay supine for 6 min to skip the transitory phase and to ensure the stabilization of the data. After this period, SpO_2_ and electrocardiogram (ECG) data were recorded for 6 min and used for calculation of the “Preliminary phase” variables. Once preliminary recording was completed, a research assistant fitted a face mask and the subject started to breathe air with the reduced O_2_ concentration. The first 5 min of hypoxia served as a stabilization period and the last 5 min were recorded and used for calculation of the “Hypoxia phase” variables. After the 10 min of hypoxia had elapsed, the mask was removed, and the subject again breathed ambient air. The first minute was used as the standardization period and 6 min of data recording followed this period. This data was used for calculation of the “Recovery phase” variables.

The altitude of the laboratory was 260 m above sea level. The normobaric hypoxia condition was equal to an altitude of 6,200 m (FiO_2_ = 9.6%) and has been used widely in the literature for intermittent hypoxic exposure (49). This condition was created using a MAG-10 system (Higher Peak, Boston, MA, United States), which simulated the lower O_2_ pressure found at high altitudes by lowering the percentage of O_2_ in the air. Subjects breathed air with a reduced O_2_ concentration via the mask from a non-rebreathing circuit with a bag acting as a reservoir.

### Oxygen saturation measurement

The SpO_2_ was measured continuously using a Nonin Avant 4000 pulse oximeter (Nonin Medical, Minneapolis, MN, United States) positioned on the right index finger. The SpO_2_ was measured at a sampling frequency of 1.0 Hz, and the average value over each phase was calculated for subsequent statistical analysis. The oxygen desaturation between Hypoxia phase and Preliminary phase was calculated as ΔSpO_2_ = SpO_2__Hypoxia_ - SpO_2__Preliminary_.

### Heart rate variability analysis

To determine the HR and HRV variables, the ECG signal was measured at a sampling frequency of 1,000 Hz using a DiANS PF8 diagnostic device (DIMEA Group, Olomouc, Czech Republic). The ECG record was examined, and all premature ventricular contractions, missing beats, and any artifacts were manually filtered. A set of 300 artifact-free subsequent R-R intervals was obtained from each phase. A spectral analysis of HRV was used to assess the ANS activity and was performed using the Fast Fourier Transform. The spectral analysis incorporated a sliding 256 points Hanning window and a Coarse-Graining Spectral Analysis algorithm (50).

Frequency domain variables included: low-frequency power (LF) calculated in the band from 0.05 to 0.15 Hz, high-frequency power (HF) calculated in the band from 0.15 to 0.50 Hz, and the LF/HF ratio. Time domain variables included: rMSSD, standard deviation of R-R intervals (SDNN), and ratio of SDNN to rMSSD. For the subsequent statistical analysis, the average HR and HRV values were calculated based on the values extracted from each phase (Preliminary, Hypoxia, and Recovery).

### Maximal oxygen uptake determination

VO_2_max, as a global indicator of physical fitness, and maximum heart rate (HRmax), were measured in normoxia during an incremental running test on the treadmill (Lode Valiant Plus, Groningen, Netherlands). The protocol consisted of a 4 min warm-up (2 min at 8 km.h^−1^ for males, and 7 km.h^−1^ for females with 0% elevation and then 2 min at the same speed at 5% elevation) followed by an increase in speed to 10 km.h^−1^ for males and 9 km.h^−1^ for females at 5% elevation for 1 min. From this point, at each minute, the speed was increased by 1 km.h^−1^, keeping elevation the same, up to 16 km.h^−1^ for males and 13 km.h^−1^ for females. The speed was then maintained and only the elevation increased by 2.5% per minute until exhaustion. Ventilation and gas exchange were recorded continuously (breath by breath) with 30 s averaging and analyzed by Blue Cherry software (Geratherm Respiratory, Bad Kissinger, Germany). The criteria for attaining VO_2_max was defined as reaching one of the following criteria: (a) respiratory exchange ratio of >1.11 (51); (b) VO_2_ plateau defined as no increase in VO_2_ in response to an increase in work rate (52). VO_2_max was considered the highest VO_2_ value in the final 30 s of the test (53). HR response was measured continuously using a chest strap (Polar, Kempele, Finland).

### Statistical analysis

All data are presented as mean ± standard deviation. Normality of distribution was checked using the Kolmogorov-Smirnov test. Skewed probability distributions of HRV indexes (LF, HF, LF/HF, SDNN, rMSSD, and SDNN/rMSSD) were corrected applying a natural logarithm (Ln). Comparisons between the sexes for anthropological and physiological characteristics were performed using the two-sample *t*-test. A 2(sex) × 3(phase) analysis of variance (ANOVA) for repeated measures was used to evaluate the effect of hypoxia on selected variables. When the ANOVA revealed a significant effect, multiple comparisons via the Fisher's LSD *post-hoc* test were performed.

To achieve results similar to Woorons et al. (17), four groups were created from our dataset. Group of females with low aerobic capacity (FL) included 7 females with the lowest VO_2_max values and group of females with high aerobic capacity (FH) included 7 females with the highest VO_2_max values. Likewise, groups of males with low (ML, *n* = 7) and high aerobic capacity (MH, *n* = 7) were created. Differences in oxygen desaturation (ΔSpO_2_ = SpO_2__Hypoxia_ - SpO_2__Preliminary_) between groups were evaluated using a 2(sex) × 2(low vs. high VO_2_max) ANOVA and Fisher's LSD *post-hoc* tests.

Effect size was calculated as standardized mean difference (Cohen's *d*) according the formula (54) $d=\frac{\left(m_{F}-m_{M}\right)}{SD_{p}}$ where *m*_*F*_ and *m*_*M*_ are means of females and males, respectively. Pooled standard deviation was calculated as follows (54) $SD_{p}=\sqrt{\frac{\left(n_{F}-1\right)\overset{k}{\underset{i=1}{\sum }}SD_{Fi}^{2}+\left(n_{M}-1\right)\overset{k}{\underset{i=1}{\sum }}SD_{Mi}^{2}}{\left(n_{F}+n_{M}-2\right)k}}$ where *n*_*F*_, *n*_*M*_ are sample sizes, SD_F_, SD_M_ are standard deviations of the females and males in *i*-th phase, and *k* = 3 is number of phases, respectively. To calculate effect sizes of pairwise differences between FL, FH, ML, and MH, pooled standard deviation was calculated as root-mean-square of standard deviations of all four groups. The following threshold values for effect size were adopted (55): <0.2 (trivial), ≥0.2 (small), ≥0.5 (medium), ≥0.8 (large).

A linear relationship between ΔSpO_2_ and normoxic VO_2_max was evaluated by means of Pearson's correlation coefficient (*r*). The null hypothesis that regression lines calculated separately for females and males would be identical was tested using a general linear model. A linear basic model contained two regression lines Δ*SpO*_2_ = *a*_*F*_ + *b*_*F*_*VO*_2_*max* and Δ*SpO*_2_ = *a*_*M*_ + *b*_*M*_*VO*_2_*max* for females and males, respectively. A linear submodel contained one regression line Δ*SpO*_2_ = *a* + *bVO*_2_*max* valid for both sexes. The magnitude of *r* was interpreted according following thresholds (55): <0.1 (trivial), ≥0.1 (small), ≥0.3 (medium), ≥0.5 (large).

For all tests, *p* < 0.05 was considered statistically significant. Statistical analyses were performed using STATISTICA 12.0 (StatSoft, Tulsa, OK, United States) and MATLAB 8.4 (MathWorks, Natick, MA, United States).

A sensitivity analysis was performed using G^*^Power version 3.1.9.2 software (56). The calculation was performed for a two-sample *t*-test for a statistical significance of 0.05, power of 0.80, and sample size of 30 and 28. The result was that the minimal detectable effect size would be *d* = 0.75. Therefore, only a large (>0.8) effect size for the population would be sufficiently detected.

## Results

Anthropological and physiological characteristics of studied groups are presented in Table 1. Importantly, females compared with males showed a significantly higher percentage of body fat, lower VO_2_max, and lower vital capacity (VC).

**Figure 2:**
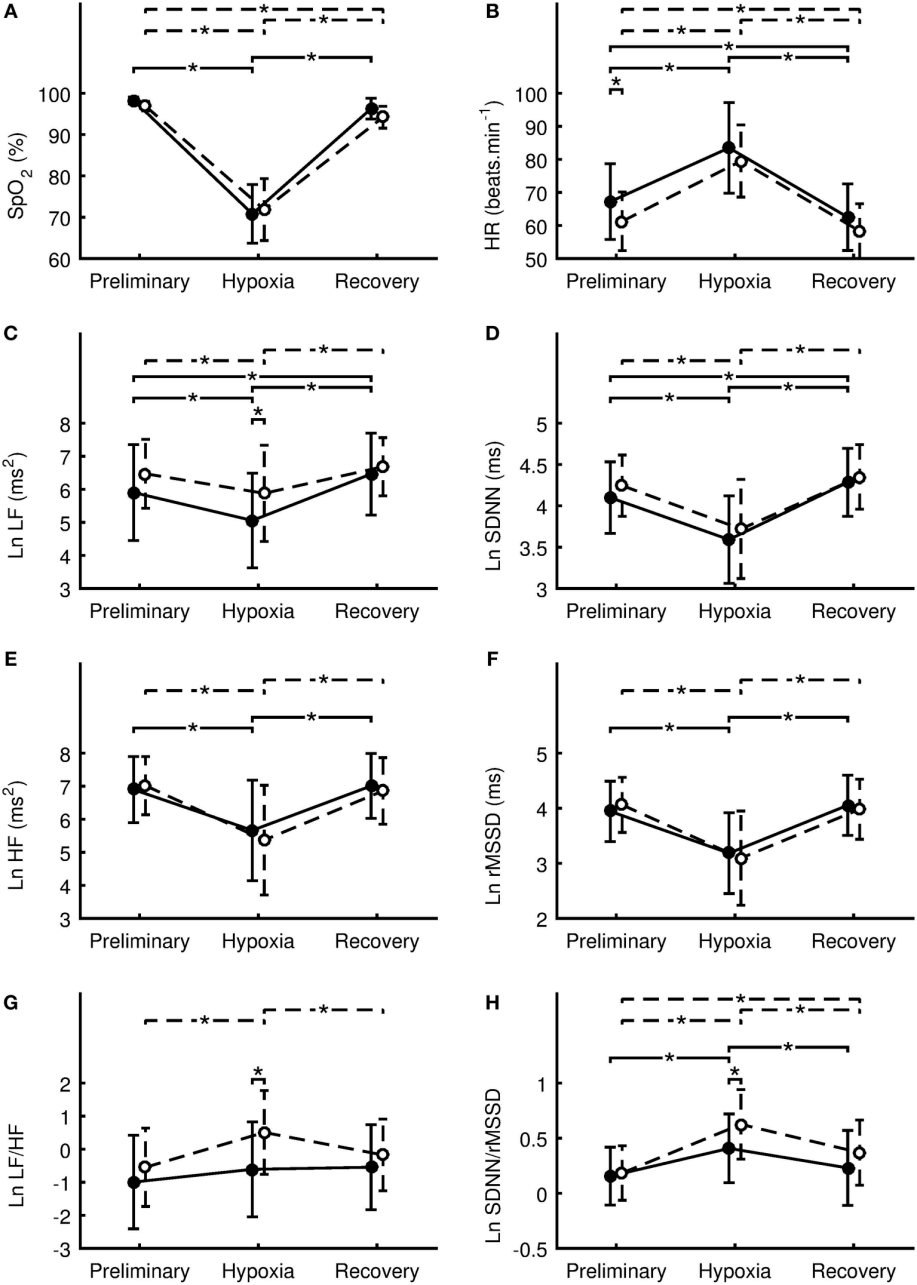
Differences between sexes in the oxygen saturation **(A)**, heart rate **(B)**, natural logarithm of low frequency power **(C)**, natural logarithm of standard deviation of RR intervals **(D)**, natural logarithm of high-frequency power **(E)**, natural logarithm of square root of the mean of the squares of the successive differences **(F)**, natural logarithm of ratio of low-frequency to high-frequency power **(G)**, natural logarithm of ratio of SDNN to rMSSD **(H)**. Values of females are denoted by filled circles and solid line. Values of males are denoted by open circles and dashed line. Values are presented as the mean ± standard deviation. Comparisons were performed by means of Fisher's LSD *post-hoc* test and only significant differences are displayed: ^*^p < 0.05.

Means and standard deviations of SpO_2_, HR, and HRV indexes in the Preliminary, Hypoxia, and Recovery phases are shown in Tables 2–**4**, respectively. Significance differences between the sexes and also between the phases are depicted in Figure 2. In the Preliminary phase, there was a significant sex difference for HR (Table 2). In the Hypoxia phase, SpO_2_ decreased significantly in both sexes (both *p* < 0.001), however, there was no significant difference between sexes during this phase (*p* = 0.376, Table 3). HR increased significantly in both sexes (both *p* < 0.001) and the sex difference that presented in the Preliminary phase was not present in the Hypoxia phase (*p* = 0.164). Vagal related indexes (Ln HF and Ln rMSSD) decreased significantly in both sexes (all *p* < 0.001) but there was no significant difference between the sexes during the Hypoxia phase (Ln HF: *p* = 0.358; Ln rMSSD: *p* = 0.590). The index of sympathovagal balance, Ln LF/HF, did not change significantly in females (*p* = 0.106) but increased significantly in males (*p* < 0.001) and there was a significant difference between sexes in the Hypoxia phase (*p* = 0.001). The time-domain surrogate for sympathovagal balance Ln SDNN/rMSSD increased significantly in both sexes (both *p* < 0.001) and there was a significant sex difference in the Hypoxia phase (*p* = 0.006). Ln LF decreased significantly in both sexes (females: *p* < 0.001; males: *p* = 0.012) and there was a significant sex difference in the Hypoxia phase (*p* = 0.016). Ln SDNN decreased significantly in both sexes (both *p* < 0.001) and there was no significant sex difference in the Hypoxia phase (*p* = 0.295).

**Table 2:** Comparison of oxygen saturation, heart rate, and HRV indexes between sexes in Preliminary phase.

	**Females**	**Males**	***p*-value**	**ES**	**ES rating**
SpO_2_ (%)	98.3 ± 0.8	96.9 ± 1.1	0.240	0.31	Small
HR (beats.min^−1^)	67 ± 11	61 ± 9	0.038	0.56	Medium
Ln LF (ms^2^)	5.9 ± 1.5	6.5 ± 1.0	0.092	−0.45	Small
Ln HF (ms^2^)	6.9 ± 1.0	7.0 ± 0.9	0.708	−0.10	Trivial
Ln LF/HF	−1.0 ± 1.4	−0.6 ± 1.2	0.188	−0.35	Small
Ln SDNN (ms)	4.10 ± 0.43	4.24 ± 0.37	0.236	−0.31	Small
Ln rMSSD (ms)	3.94 ± 0.55	4.06 ± 0.50	0.486	−0.18	Trivial
Ln SDNN/rMSSD	0.16 ± 0.26	0.18 ± 0.25	0.716	−0.10	Trivial
LF (ms^2^)	1,003 ± 1,655	1,148 ± 1,760		
HF (ms^2^)	1,556 ± 1,534	1,547 ± 1,249		
LF/HF	1.2 ± 2.6	1.2 ± 2.0		
SDNN (ms)	66 ± 28	75 ± 29		
rMSSD (ms)	59 ± 30	65 ± 32		
SDNN/rMSSD	1.21 ± 0.31	1.24 ± 0.34		

Values are presented as mean ± SD.

*ES, effect size (Cohen's d); SpO_2_, oxygen saturation; HR, heart rate; Ln, natural logarithm; LF, low-frequency power; HF, high-frequency power; LF/HF, ratio of low-frequency to high-frequency power; SDNN, standard deviation of RR intervals; rMSSD, square root of the mean of the squares of the successive differences; SDNN/rMSSD, ratio of SDNN to rMSSD*.

**Table 3:** Comparison of oxygen saturation, heart rate, and HRV indexes between sexes in Hypoxia phase.

	**Females**	**Males**	***p*-value**	**ES**	**ES rating**
SpO_2_ (%)	70.8 ± 7.1	71.9 ± 7.5	0.376	−0.23	Small
HR (beats.min^−1^)	83 ± 14	80 ± 11	0.164	0.37	Small
Ln LF (ms^2^)	5.1 ± 1.4	5.9 ± 1.5	0.016	−0.65	Medium
Ln HF (ms^2^)	5.7 ± 1.5	5.4 ± 1.7	0.358	0.24	Small
Ln LF/HF	−0.6 ± 1.4	0.5 ± 1.3	0.001	−0.87	Large
Ln SDNN (ms)	3.59 ± 0.53	3.72 ± 0.60	0.295	−0.28	Small
Ln rMSSD (ms)	3.18 ± 0.73	3.09 ± 0.86	0.590	0.14	Trivial
Ln SDNN/rMSSD	0.41 ± 0.31	0.63 ± 0.32	0.006	−0.73	Medium
LF (ms^2^)	501 ± 1, 268	1,011 ± 1,628		
HF (ms^2^)	656 ± 650	762 ± 1, 297		
LF/HF	1.5 ± 2.6	3.4 ± 5.3		
SDNN (ms)	42 ± 22	50 ± 35		
rMSSD (ms)	31 ± 22	33 ± 33		
SDNN/rMSSD	1.58 ± 0.51	1.96 ± 0.59		

Values are presented as mean ± SD.

*ES, effect size (Cohen's d); SpO_2_, oxygen saturation; HR, heart rate; Ln, natural logarithm; LF, low-frequency power; HF, high-frequency power; LF/HF, ratio of low-frequency to high-frequency power; SDNN, standard deviation of RR intervals; rMSSD, square root of the mean of the squares of the successive differences; SDNN/rMSSD, ratio of SDNN to rMSSD*.

During the Recovery phase, there was no significant difference between the sexes (Table 4). However, differences in dynamics between Preliminary and Recovery phases were found as follow. In females, SpO_2_ recovered during the Recovery phase to value not significantly (*p* > 0.064) different from the Preliminary value, however, SpO_2_ in males did not fully recover and remain significantly (*p* = 0.017) decreased compared to the Preliminary value. HR during the Recovery phase decreased significantly in both sexes (females: *p* < 0.001; males: *p* = 0.011) below the Preliminary values. In both sexes, vagal related indexes (Ln HF and Ln rMSSD) recovered to values not significantly (all *p* > 0.263) different from the Preliminary values. In both sexes, Ln LF/HF recovered to values not significantly (females: *p* = 0.060; males: *p* = 0.131) different from the Preliminary values. Ln SDNN/rMSSD in females recovered to value not significantly (*p* = 0.122) different from the Preliminary value, however, the index in males did not fully recover and remain significantly (*p* < 0.001) elevated compared to the Preliminary value. In males, indexes Ln LF and Ln SDNN recovered to values not significantly (Ln LF: *p* = 0.361; Ln SDNN: *p* = 0.211) different from the Preliminary values. However, in females, the indexes during the Recovery phase increased significantly (Ln LF: *p* = 0.014; Ln SDNN: *p* = 0.025) above the Preliminary values.

**Table 4:** Comparison of oxygen saturation, heart rate, and HRV indexes between sexes in Recovery phase.

	**Females**	**Males**	***p*-value**	**ES**	**ES rating**
SpO_2_ (%)	96.3 ± 2.5	94.2 ± 2.6	0.081	0.46	Small
HR (beats.min^−1^)	63 ± 10	58 ± 8	0.140	0.40	Small
Ln LF (ms^2^)	6.5 ± 1.2	6.7 ± 0.9	0.510	−0.17	Trivial
Ln HF (ms^2^)	7.0 ± 1.0	6.9 ± 1.0	0.637	0.12	Trivial
Ln LF/HF	−0.5 ± 1.3	−0.2 ± 1.1	0.275	−0.29	Small
Ln SDNN (ms)	4.29 ± 0.41	4.35 ± 0.39	0.592	−0.14	Trivial
Ln rMSSD (ms)	4.05 ± 0.55	3.98 ± 0.54	0.660	0.12	Trivial
Ln SDNN/rMSSD	0.23 ± 0.34	0.37 ± 0.30	0.079	−0.47	Small
LF (ms^2^)	1,270 ± 1,655	1,141 ± 1,009		
HF (ms^2^)	1,671 ± 1,608	1,536 ± 1,578		
LF/HF	1.3 ± 2.6	1.5 ± 1.8		
SDNN (ms)	79 ± 31	83 ± 31		
rMSSD (ms)	66 ± 32	62 ± 34		
SDNN/rMSSD	1.34 ± 0.52	1.51 ± 0.44		

Values are presented as mean ± SD.

*ES, effect size (Cohen's d); SpO_2_, oxygen saturation; HR, heart rate; Ln, natural logarithm; LF, low-frequency power; HF, high-frequency power; LF/HF, ratio of low-frequency to high-frequency power; SDNN, standard deviation of RR intervals; rMSSD, square root of the mean of the squares of the successive differences; SDNN/rMSSD, ratio of SDNN to rMSSD*.

**Figure 3:**
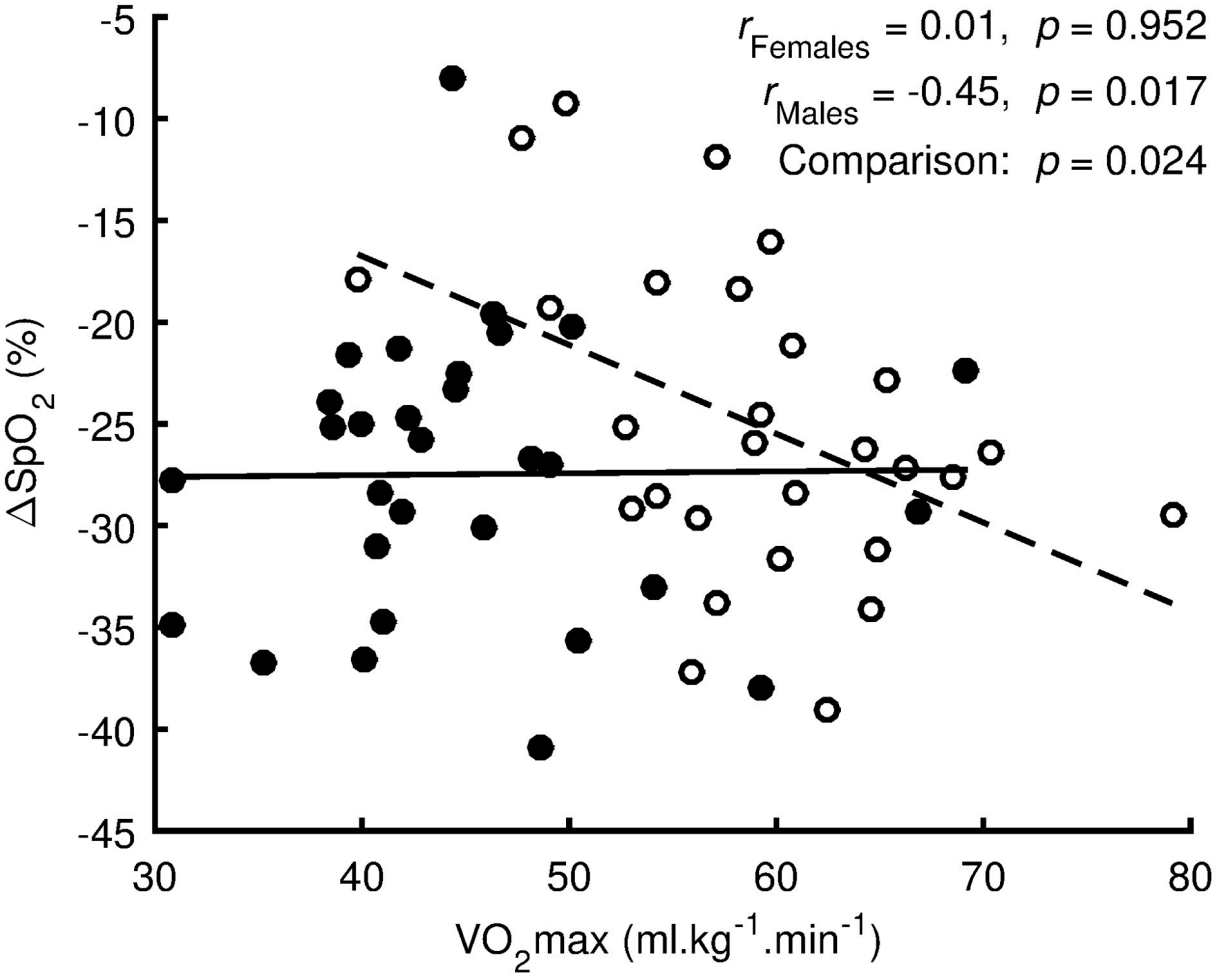
Regression analysis between oxygen desaturation (ΔSpO_2_ = SpO_2__Hypoxia_-SpO_2__Preliminary_) and the maximal oxygen uptake (VO_2_max) measured in normoxia. Values of females are denoted by filled circles and solid line. Values of males are denoted by open circles and dashed line.

Regression analysis (Figure 3) showed no linear relationship between ΔSpO_2_ and VO_2_max (*p* = 0.952, *r* = 0.01, trivial effect) for females. However, there was a significant linear relationship (*p* = 0.017, *r* = −0.45, medium effect) for males. The difference between regression lines for females and males was significant (*p* = 0.024).

**Figure 4:**
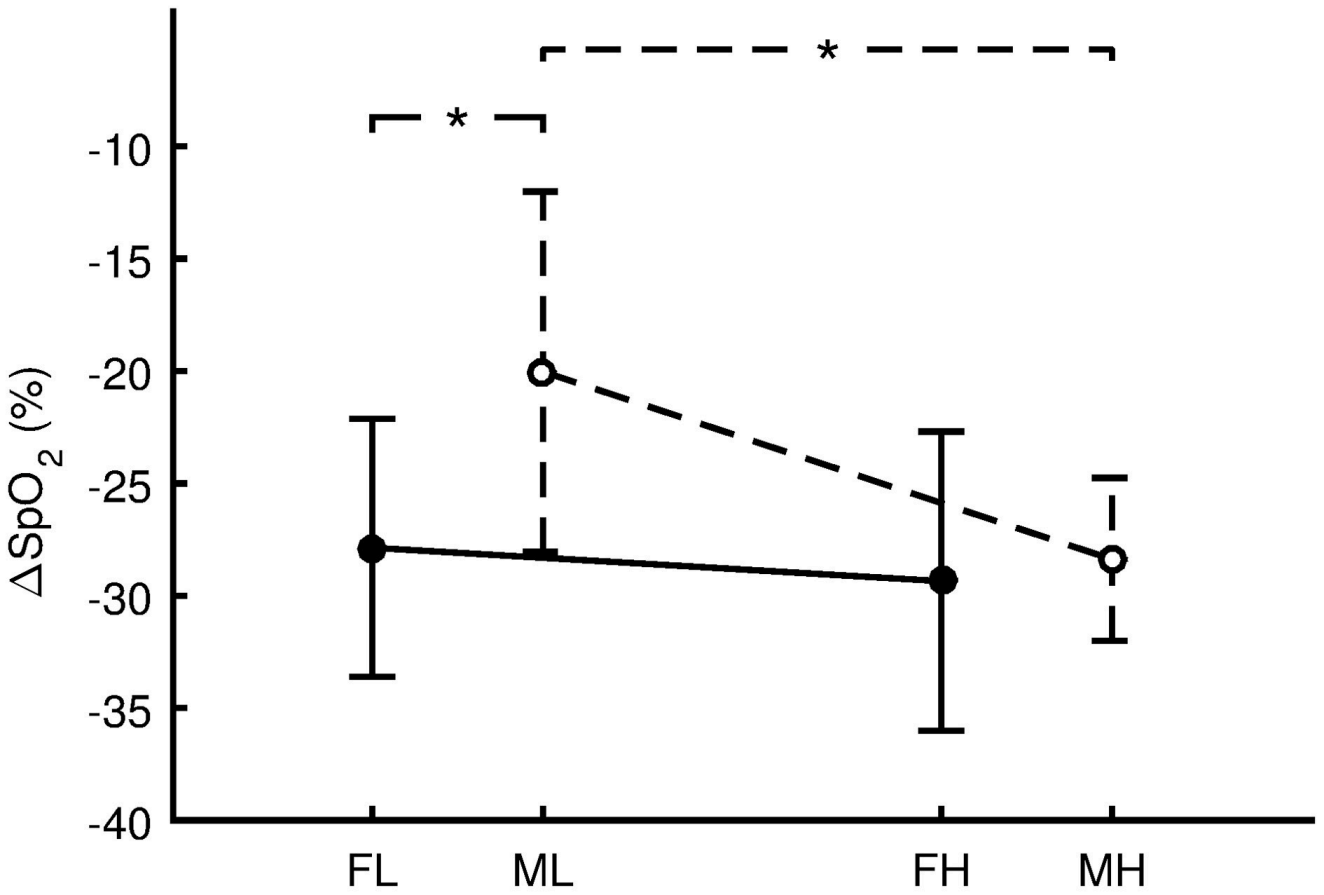
Differences in the oxygen desaturation (ΔSpO_2_ = SpO_2__Hypoxia_ - SpO_2__Preliminary_) between females with low aerobic capacity (FL, *n* = 7), males with low aerobic capacity (ML, *n* = 7), females with high aerobic capacity (FH, *n* = 7), and males with high aerobic capacity (MH, *n* = 7). Values are presented as the mean ± standard deviation. Comparisons were performed by means of Fisher's LSD *post-hoc* test and only significant differences are displayed: ^*^p < 0.05.

For the females, VO_2_max was 36.2 ± 3.9 ml.kg^−1^.min^−1^ for FL and 57.0 ± 8.3 ml.kg^−1^.min^−1^ for FH. There was no significant difference (Figure 4, *p* = 0.659, *d* = 0.24, small effect) in ΔSpO_2_ between FL (−27.9 ± 5.7%) and FH (−29.4 ± 6.7%). For the males, VO_2_max was 49.5 ± 4.9 ml.kg^−1^.min^−1^ for ML and 68.4 ± 5.2 ml.kg^−1^.min^−1^ for MH. ΔSpO_2_ for ML (−20.0 ± 8.0%) was significantly less steep (*p* = 0.019, *d* = 1.34, large effect) compared with MH (−28.4 ± 3.6%). Regarding sex differences, ΔSpO_2_ for ML was significantly less steep (*p* = 0.027, *d* = 1.26, large effect) compared with FL. However, the difference between MH and FH was not significant (*p* = 0.773, d = 0.16, trivial effect).

## Discussion

The primary purpose of this study was to assess whether the autonomic activity and SpO_2_ responses to 10 min resting normobaric hypoxia (FiO_2_ = 9.6%, simulated altitude ~6,200 m) were similar in age matched males and females. The secondary aim was to determine if there were sex-related differences in the association between VO_2_max and the SpO_2_ response to hypoxia. The primary novel findings of the study were as follows: (a) there were significant sex differences in autonomic cardiac control during the hypoxia period, with the males displaying a similar desaturation level but a relatively higher sympathetic stimulation (LF/HF) compared with the females; (b) there was a significantly (*p* = 0.024) different association between ΔSpO_2_ and normoxic VO_2_max level between males and females during resting normobaric hypoxia exposure. Specifically, the ΔSpO_2_ response to hypoxia indicated no association to VO_2_max level in females but it was moderately correlated with VO_2_max in males, and (c) males who exhibited lower aerobic capacity demonstrated smaller desaturation in resting hypoxia compared with females with low aerobic capacity.

Based on our statistical analysis in the Preliminary phase, there were no significant differences between males and females in either SpO_2_ nor in HRV variables, except lower resting HR in males. In support of this finding, it was previously demonstrated that males have a lower resting HR compared with females, due to larger cardiac chamber size and higher resting stroke volume in males (57, 58). Moreover, a lower resting HR is widely accepted as typical sign of cardiovascular system adaptation to endurance training (59, 60), and the males demonstrated a significantly higher aerobic performance compared with females in the present study. In this study, SpO_2_ and vagally-related variables (HF and rMSSD) dropped in similar fashion in both groups with no differences between groups during the 10 min of resting hypoxia exposure. However, despite similar HRV LF and LF/HF variables during the Preliminary phase, LF was significantly lower in females compared with males during hypoxia. Consequently, the increased LF/HF ratio, considered as sympathovagal balance index (31), indicated a relatively higher sympathetic involvement in cardiovascular control during hypoxia in males compared with females. A similar response to the LF/HF ratio during hypoxia was seen in the SDNN/rMSSD ratio, that is currently accepted as an alternative index of sympathovagal balance, derived from time domain analysis by some authors (34, 61, 62). The functional changes found in autonomic cardiac activity were also reflected in the higher HR response during hypoxia in males compared with females.

Wadhwa et al. (40), who assessed effect of sex-related differences in HRV in response to intermittent normobaric hypoxia exposure (FiO_2_ = 8.0%), demonstrated a similar decline in vagal activity when the final and initial hypoxic periods were compared. However, a more pronounced reduction in vagal activity together with the progressive shift of the sympathovagal balance to sympathetic dominance was evident in males but not in females. In a recently published meta-analysis on gender related HRV levels in resting normoxia, it was shown that although healthy females have a lower mean R-R interval together with lower global autonomic cardiac activity compared with age matched males at rest, females maintain significantly greater HF and less LF power that is further reflected by a lower LF/HF ratio. This was proposed as a potential cardio-protective regulatory effect of the relatively higher cardiac vagal involvement in cardiovascular control that could be related to ovarian hormones and/or oxytocin (39). Based on our results, maintaining balance between vagal and sympathetic activity during resting normoxia, and within short-term acute normobaric hypoxia, seem to be more important for females compared with males irrespective of whether global autonomic cardiac activity is lower in females during hypoxia exposure. Our findings may reflect an estrogen related attenuation in sympathoadrenal stress response (63) that may provide a protective effect on the cardiovascular system when under environmental stress such as hypoxia. This proposal is indirectly supported by Huikuri et al. (64) who demonstrated that post-menopausal females with estrogen replacement therapy showed significantly higher baroreflex sensitivity and total HRV compared with age-matched females without hormone treatment. Moreover, when sex-related responses to upright posture were compared, the females had a significantly attenuated increase in HR, and a smaller decrease in the HF component. Boos et al. (41) demonstrated a significant reduction in vagal activity due to an increasing hypobaric hypoxia during ascent up to an altitude of 5,140 m amongst males and females. Compared with the present study, at altitude, the males exhibit significantly higher vagally-related HRV variables and higher global HRV than females, while no significant differences in sympathovagal balance were reported between genders. In this case, a lower HRV in females especially at altitudes of 4,600 and 5,140 m may have been associated with elevated fatigue, potentially due to a lower cardiorespiratory fitness, during the strenuous ascents in the females. To explain these different findings between Boos et al. (41) and the present study, it is proposed that our results specifically reflect an acute autonomic cardiac response to hypoxia in unacclimatized persons, whereas Boos et al. (41) described chronic changes in autonomic cardiac activity which may mirror an influence of several factors, for instance, an individual course of acclimatization to altitude.

Despite no significant (*p* = 0.081) sex-related difference in SpO_2_ being found during recovery in the present study, there was a significant delay in SpO_2_ return to baseline in the males compared with the females, whose SpO_2_ returned to preliminary values rapidly once hypoxia was removed. We propose that for the males, the slower SpO_2_ return to baseline during the recovery phase was because of persistent sympathetic dominance during recovery compared with the preliminary phase. Our results support the findings of Jones et al. (65), who assessed sympathetic response via muscle sympathetic nerve activity during 15 min of normobaric hypoxia (FiO_2_ = 10%) and following 10 min recovery. They reported that sex appears to contribute to the interindividual variability in the sympathetic cardiovascular response to a hypoxia environment. Females exhibited a decline in sympathetic activity in the first minute of recovery post-hypoxia, compared with males, where the decrease in sympathetic activity post-hypoxia took up to 6 min.

Following the suppression in autonomic regulation of cardiac activity during the hypoxia stress in the present study, there appeared to be a “supercompensation phenomenon” in overall autonomic cardiac activity during the recovery phase, in the females, but not in the males. However, there was a significant decrease in the mean HR compared to baseline values in both sexes. In light of this, Roche et al. (66) who assessed change in both baroreflex sensitivity (BRS) and HRV in response to 15 min hypoxia, (FiO_2_ = 11%) reported that a relative bradycardia during 20 min normoxic recovery was modulated throughout improved vagal activity together with transitory significant overactivity of the spontaneous BRS. Unfortunately, Roche et al. (66) did not examine sex differences. In contrast to Roche et al. (66), Halliwill et al. (67) reported that acute exposure to hypoxia reset baroreflex control of both HR and sympathetic activity to higher pressures without changes in BRS.

To the best of our knowledge, this is the first study to show a significant (*p* = 0.024) sex-related difference in ΔSpO_2_ is related to normoxic VO_2_max level between males and females during short-term, acute, resting normobaric hypoxia exposure. Previously, Macoun et al. (9), who assessed ΔSpO_2_ response in relation to individual aerobic fitness during similar hypoxia conditions (10 min; FiO_2_ = 9.6%; simulated altitude ~6,200 m), reported a negative correlation between ΔSpO_2_ and VO_2_max (*r* = −0.45; *p* = 0.017) in males. A negative effect of a higher VO_2_max on desaturation, especially during exercise, in endurance well-trained, males has previously been reported (8, 16, 17, 20). Researchers ascribed this relationship to a number of factors, including, possible blunted chemoreceptor sensitivity (68), resulting in an insufficient ventilatory response (relative hypoventilation) to severe hypoxia in well-trained endurance athletes at rest (17), but especially during exercise in moderate, normobaric hypoxia (17). In contrast to the males, based on our regression analysis, there was no relationship (*r* = 0.01, *p* = 0.952) between VO_2_max level achieved in a normoxic environment and the ΔSpO_2_ response during acute resting, normobaric, hypoxia exposure in our cohort of females. Our results are supported by previously published study (18), where authors reported that there were no significant differences between aerobically well-trained and sedentary females for resting SpO_2_ at different levels of normobaric, hypoxia environment (FiO_2_ = 0.187, 0.154, and 0.117). However, once the SpO_2_ response was assessed during exercise under hypoxia conditions, a similar influence of higher VO_2_max on desaturation level was identified in aerobically well-trained females, probably due to diffusion limitation (18). Former studies have repeatedly shown that the AHVR, a vital body response for homeostatic SpO_2_ adjustment during hypoxia (12), depends on the VO_2_max level in males (8, 16, 17), whereas in females the factors that contribute to AHVR during hypoxia have yet to be clarified (69, 70). It has been shown that AHVR varies depending on the menstrual cycle phase (71). For instance, progesterone, which peaks during the luteal phase (72), was found to stimulate AHVR (73) via central (74) and peripheral (75) receptor induced-mechanisms. Based on self-reports, all our female subjects participated in the hypoxia exposure during the follicular phase, when, according to Guenette et al. (70), AHVR is not different between trained and untrained females.

The lack of correlation between VO_2_max and ΔSpO_2_ in females, despite the comparable desaturation level among our males and females at similar simulated altitudes, is a novel finding. The result implies that whilst males with lower aerobic capacity demonstrated less desaturation in acute resting hypoxia, the females, irrespective of their aerobic capacity had low SpO_2_. Therefore, while lower aerobic capacity in males seemed to be protective, this is not the case for females. A possible explanation for this finding is that these females exhibit insufficient AHVR because of an altered sympathoadrenal system activation that may have been modulated by the level of estrogen (39). In this context, Lusina et al. (76) reported, that following intermittent hypoxic training, the rise in sympathetic activity was strongly related to the change in AHVR (*r* = 0.79, *p* < 0.05) suggesting that sympathetic and ventilatory responses may have a common central control. A second explanation for the sex-related difference was discussed in a review by Harms (71) who showed that females exhibit lower lung diffusion capacity compared to age- and height-matched males due to both smaller diffusion surfaces (77), and smaller airways diameter relative to lung size (78). In case of similar ventilation, females may exhibit lower SpO_2_ compared to males. A further explanation may be related to potentially lower hemoglobin levels in the females. Low hemoglobin is associated with an increased level of 2,3-diphosphoglycerate (DPG), due to the decrease in oxygen carrying capacity (79). This results in a shift in oxyhemoglobin dissociation curve to the right, decreasing the affinity of oxygen to the hemoglobin, and reducing the SpO_2_ (79). This shift is exaggerated at high altitude and females may be more sensitive to this shift, irrespective of VO_2_max. Supporting this idea is research (80) demonstrating that oxyhemoglobin dissociation curve is different (at the same temperature and pH) in the two sexes and that females present less hemoglobin affinity for oxygen, with 2,3-DPG levels 2 mmol/g of hemoglobin higher compared with males. However, these reasons are currently speculative and further study is required with the measurement of additional physiological data that could help explain the mechanism behind this sex difference.

From a practical perspective, we propose that lower aerobic capacity may represent a temporary advantage, particularly in males, who are performing a rapid, passive ascent to high altitude without previous hypoxia exposure. In contrast, males with a high aerobic capacity (>65 ml.kg^−1^.min^−1^) and females in the follicular phase of the menstrual cycle, may be more vulnerable to a higher desaturation during an acute hypoxia exposure, and consequently, these subjects may be considered to be at a greater risk of developing AMS. These suggestions are supported by Karinen et al. (15) who reported that subjects who manifested AMS symptoms exhibited both a greater decline in SpO_2_ in hypoxia and a higher VO_2_max compared with subjects who were free from AMS symptoms. Similarly, Álvarez-Herms et al. (1) recently showed that higher appearance in AMS symptoms was scored by professional compared to amateur endurance-trained athletes who performed an altitude training camp probably due to higher training doses in professional athletes.

In individuals previously highly sensitive to acute hypoxia exposure, a smaller decrease in both SpO_2_ and HRV was found after specialized pre-acclimatization using normobaric, hypoxic, intermittent training (6). Thus, in order to avoid a progressive desaturation and potentially AMS when exposed to altitude, hypoxic acclimation training may represent a promising strategy for both females and aerobically fit males who plan to use passive transport to altitude without following staged acclimatization that occurs during active ascent to altitude.

A main limitation of this study was that, during hypoxia, subjects wore a face mask, and therefore, it was not possible to appropriately assess AHVR that typically occurs within 5 min of hypoxia exposure (12). A knowledge of the AHVR may have helped explain the VO_2_max vs. SpO_2_ association difference between males and females. The diagnostics system (DiANS PF8) used to determine HRV changes during the different study phases only provided data about changes in BF. Future research should include complete ventilatory response assessment (e.g., minute ventilation, dead space analysis, PetCO_2_) and measurements of A-a gradient calculation, hemoglobin concentration, pO_2_, 2,3-DPG levels, and sex hormone levels. In addition, continual monitoring of blood pressure and/or BRS (66, 67) may be beneficial for providing a more complex view of the autonomic regulation of the cardiopulmonary system during a resting acute, normobaric hypoxia. Another limitation of this study was that it could be considered as underpowered based on the sensitivity analysis.

## Conclusion

Despite finding similar oxygen desaturation levels and vagal withdrawal between genders during hypoxia; females demonstrated a relatively lower sympathetic response to the resting hypoxia exposure, compared with males. Delayed return in SpO_2_ to its baseline during recovery after hypoxia exposure may be because of prolonged sympathetic stimulation in the males, but not the females. Moreover, there was a sex-related difference in the resting, acute, hypoxia response, relating to the association between SpO_2_ levels, and maximal aerobic capacity. Specifically, resting VO_2_max in females was not associated with resting desaturation levels, whereas in males, VO_2_max was associated with the SpO_2_ response.

## Data availability

The raw data supporting the conclusions of this manuscript will be made available by the authors, without undue reservation, to any qualified researcher.

## Author contributions

MB contributed to the design of the study. MB and JK performed data collection. JK performed the statistical analysis. MB wrote the first draft of the manuscript. MB, JK, and AM wrote sections of the manuscript. All authors contributed to manuscript revision, read, and approved the submitted version.

### Conflict of interest statement

The authors declare that the research was conducted in the absence of any commercial or financial relationships that could be construed as a potential conflict of interest.

## References

[1] Álvarez-Herms J Julià-Sánchez S Hamlin MJ Corbi F Pagès T Viscor G. Popularity of hypoxic training methods for endurance-based professional and amateur athletes. Physiol Behav. (2015) 143:35–8. 10.1016/j.physbeh.2015.02.020 25698671

[2] Bonetti D Hopkins W. Se-level exercise performance following adaptation to hypoxia: a meta analysis. Sports Med. (2009) 39:107–27. 10.2165/00007256-200939020-00002 19203133

[3] Wilber RL. Application of altitude/hypoxic training by elite athletes. Med Sci Sport Exerc. (2007) 39:1610–24. 10.1249/mss.0b013e3180de49e6 17805095

[4] Chen YC Lin FC Shiao GM Chang SC. Effect of rapid ascent to high altitude on autonomic cardiovascular modulation. Am J Med Sci. (2008) 336:248–53. 10.1097/MAJ.0b013e3181629a32 18794620

[5] Sutherland A Freer J Evans L Dolci A Crotti M Macdonald JH. MEDEX 2015: heart rate variability predicts development of acute mountain sickness. High Alt Med Biol. (2017) 18:199–208. 10.1089/ham.2016.0145 28418725

[6] Bobyleva OV Glazachev OS. Changes in autonomic response and resistance to acute graded hypoxia during intermittent hypoxic training. Fiziol Cheloveka (2007) 33:81–9. 10.1134/S0362119707020107 17486993

[7] Botek M Krejčí J De Smet S Gába A McKune AJ. Heart rate variability and arterial oxygen saturation response during extreme normobaric hypoxia. Auton Neurosci. (2015) 190:40–5. 10.1016/j.autneu.2015.04.001 25907329

[8] Chapman RF. The individual response to training and competition at altitude. Br J Sports Med. (2013) 47 (Suppl. 1):i40–4. 10.1136/bjsports-2013-092837 24282206 PMC3903142

[9] Macoun T Botek M Krejčí J McKune AJ Vagal activity and oxygen saturation response to hypoxia: effects of aerobic fitness and rating of hypoxia tolerance. Acta Gymnica (2017) 47:112–21. 10.5507/ag.2017.014

[10] Oliveira ALMB Philippe de Azeredo R Gonçalves TR Pedro Paulo da Silva S Effects of hypoxia on heart rate variability in healthy individuals: a systematic review. Int J Cardiovasc Sci. (2017) 30:251–61. 10.5935/2359-4802.20170035

[11] Rowell LB Johnson DG Chase PB Comess KA Seals DR Hypoxemia raises muscle sympathetic activity but not norepinephrine in resting humans. J Appl Physiol. (1989) 66:1736–43. 10.1152/jappl.1989.66.4.1736 2732164

[12] Hainsworth R Drinkhill MJ Rivera-Chira M. The autonomic nervous system at high altitude. Clin Auton Res. (2007) 17:13–9. 10.1007/s10286-006-0395-7 17264976 PMC1797062

[13] Ainslie PN Poulin MJ. Ventilatory, cerebrovascular, and cardiovascular interactions in acute hypoxia: regulation by carbon dioxide. J Appl Physiol. (2004) 97:149–59. 10.1152/japplphysiol.01385.2003 15004003

[14] Kawakami Y Yoshikawa T Shida A Asanuma Y. Relationship between hypoxic and hypercapnic ventilatory responses in man. Jpn J Physiol. (1981) 31:357–68. 10.2170/jjphysiol.31.357 7300042

[15] Karinen HM Peltonen JE Kähönen M Tikkanen HO. Prediction of acute mountain sickness by monitoring arterial oxygen saturation during ascent. High Alt Med Biol. (2010) 11:325–32. 10.1089/ham.2009.1060 21190501

[16] Gore CJ Hahn AG Scroop GC Watson DB Norton KI Wood RJ. Increased arterial desaturation in trained cyclists during maximal exercise at 580 m altitude. J Appl Physiol. (1996) 80:2204–10. 10.1152/jappl.1996.80.6.2204 8806931

[17] Woorons X Mollard P Pichon A Lamberto C Duvallet A Richalet JP. Moderate exercise in hypoxia induces a greater arterial desaturation in trained than untrained men. Scand J Med Sci Sport (2007) 17:431–6. 10.1111/j.1600-0838.2006.00577.x 16805783

[18] Woorons X Mollard P Lamberto C Letournel M Richalet J-P. Effect of acute hypoxia on maximal exercise in trained and sedentary women. Med Sci Sports Exerc. (2005) 37:147–54. 10.1249/01.MSS.0000150020.25153.34 15632681

[19] Byrne-Quinn E Weil JV Sodal IE Filley GF Grover RF. Ventilatory control in the athlete. J Appl Physiol. (1971) 30:91–8. 10.1152/jappl.1971.30.1.91 5538798

[20] Dempsey JA Hanson PG Henderson KS. Exercise-induced arterial hypoxaemia in healthy human subjects at sea level. J Physiol. (1984) 355:161–75. 10.1113/jphysiol.1984.sp015412 6436475 PMC1193484

[21] Mazzeo RS. Physiological responses to exercise. Sport Med. (2008) 38:1–8. 10.2165/00007256-200838010-00001 18081363

[22] Chacaroun S Borowik A Morrison SA Baillieul S Flore P Doutreleau S. Physiological responses to two hypoxic conditioning strategies in healthy subjects. Front Physiol. (2017) 7:675. 10.3389/fphys.2016.00675 28119623 PMC5222853

[23] Serebrovskaya TV. Intermittent hypoxia research in the former soviet union and the commonwealth of independent States: history and review of the concept and selected applications. High Alt Med Biol. (2002) 3:205–21. 10.1089/15270290260131939 12162864

[24] Mounier R Pialoux V Schmitt L Richalet J-P Robach P Coudert J. Effects of acute hypoxia tests on blood markers in high-level endurance athletes. Eur J Appl Physiol. (2009) 106:713–20. 10.1007/s00421-009-1072-z 19430946

[25] Palazon A Goldrath AW Nizet V Johnson RS. HIF transcription factors, inflammation, and immunity. Immunity (2014) 41:518–28. 10.1016/j.immuni.2014.09.008 25367569 PMC4346319

[26] Akselrod S Gordon D Ubel FA Shannon DC Berger AC Cohen RJ. Power spectrum analysis of heart rate fluctuation: a quantitative probe of beat-to-beat cardiovascular control. Science (1981) 213:220–2. 10.1126/science.6166045 6166045

[27] Aubert AE Seps B Beckers F. Heart rate variability in athletes. Sports Med. (2003) 33:889–919. 10.2165/00007256-200333120-00003 12974657

[28] Yasuma F Hayano J-I. Respiratory sinus arrhythmia: why does the heartbeat synchronize with respiratory rhythm? Chest (2004) 125:683–90. 10.1378/chest.125.2.683 14769752

[29] Goldstein DS Bentho O Park MY Sharabi Y Low-frequency power of heart rate variability is not a measure of cardiac sympathetic tone but may be a measure of modulation of cardiac autonomic outflows by baroreflexes. Exp Physiol. (2011) 96:1255–61. 10.1113/expphysiol.2010.056259 21890520 PMC3224799

[30] Task Force of the European Society of Cardiology and the North American Society of Pacing and Electrophysiology Heart rate variability. Standards of measurement, physiological interpretation, and clinical use. Eur Heart J Engl. (1996) 17:354–81. 10.1093/oxfordjournals.eurheartj.a014868 8737210

[31] Malliani A Pagani M Lombardi F Cerutti S. Cardiovascular neural regulation explored in the frequency domain. Circulation (1991) 84:482–92. 10.1161/01.CIR.84.2.482 1860193

[32] Ori Z Monir G Weiss J Sayhouni X Singer DH. Heart rate variability. Frequency domain analysis. Cardiol Clin. (1992) 10:499–537. 10.1016/S0733-8651(18)30231-5 1504981

[33] Iwasaki KI Ogawa Y Aoki K Saitoh T Otsubo A Shibata S. Cardiovascular regulation response to hypoxia during stepwise decreases from 21% to 15% inhaled oxygen. Aviat Space Environ Med. (2006) 77:1015–9. 17042245

[34] Krejčí J Botek M McKune AJ. Dynamics of the heart rate variability and oxygen saturation response to acute normobaric hypoxia within the first 10 min of exposure. Clin Physiol Funct Imaging (2018) 38:56–62. 10.1111/cpf.12381 27380961

[35] Povea C Schmitt L Brugniaux J Nicolet G Richalet J-P Fouillot J-P. Effects of intermittent hypoxia on heart rate variability during rest and exercise. High Alt Med Biol. (2005) 6:215–25. 10.1089/ham.2005.6.215 16185139

[36] Buch AN Coote JH Townend JN. Mortality, cardiac vagal control and physical training–what's the link? Exp Physiol. (2002) 87:423–35. 10.1111/j.1469-445X.2002.tb00055.x 12392106

[37] Pal GK Adithan C Dutta TK Pal P Nanda N Lalitha V. Association of hypertension status and cardiovascular risks with sympathovagal imbalance in first degree relatives of type 2 diabetics. J Diabetes Invest. (2014) 5:449–55. 10.1111/jdi.12166 25411606 PMC4210069

[38] Billman GE. Cardiac autonomic neural remodeling and susceptibility to sudden cardiac death: effect of endurance exercise training. Am J Physiol Heart Circ Physiol. (2009) 297:H1171–93. 10.1152/ajpheart.00534.2009 19684184

[39] Koenig J Thayer JF. Sex differences in healthy human heart rate variability: a meta-analysis. Neurosci Biobehav Rev. (2016) 64:288–310. 10.1016/j.neubiorev.2016.03.007 26964804

[40] Wadhwa H Gradinaru C Gates GJ Badr MS Mateika JH. Impact of intermittent hypoxia on long-term facilitation of minute ventilation and heart rate variability in men and women: do sex differences exist? J Appl Physiol. (2008) 104:1625–33. 10.1152/japplphysiol.01273.2007 18403450 PMC2569839

[41] Boos CJ Vincent E Mellor A O'Hara J Newman C Cruttenden R. The effect of sex on heart rate variability at high altitude. Med Sci Sports Exerc. (2017) 49:2562–9. 10.1249/MSS.0000000000001384 28731986

[42] Boos CJ Mellor A O'Hara JP Tsakirides C Woods DR. The effects of sex on cardiopulmonary responses to acute normobaric hypoxia. High Alt Med Biol. (2016) 17:108–15. 10.1089/ham.2015.0114 27008376

[43] Brown TE Beightol LA Koh J Eckberg DL. Important influence of respiration on human R-R interval power spectra is largely ignored. J Appl Physiol. (1993) 75:2310–7. 10.1152/jappl.1993.75.5.2310 8307890

[44] Hirsch JA Bishop B. Respiratory sinus arrhythmia in humans: how breathing pattern modulates heart rate. Am J Physiol. (1981) 241:620–9. 7315987 10.1152/ajpheart.1981.241.4.H620

[45] Sasaki K Maruyama R. Consciously controlled breathing decreases the high-frequency component of heart rate variability by inhibiting cardiac parasympathetic nerve activity. Tohoku J Exp Med. (2014) 233:155–63. 10.1620/tjem.233.155 24965685

[46] Ito S Sasano H Sasano N Hayano J Fisher JA Katsuya H. Vagal nerve activity contributes to improve the efficiency of pulmonary gas exchange in hypoxic humans. Exp Physiol. (2006) 91:935–41. 10.1113/expphysiol.2006.034421 16809376

[47] Patwardhan AR Vallurupalli S Evans JM Bruce EN Knapp CF. Override of spontaneous respiratory pattern generator reduces cardiovascular parasympathetic influence. J Appl Physiol. (1995) 79:1048–54. 10.1152/jappl.1995.79.3.1048 8567501

[48] Tada Y Yoshizaki T Tomata Y Yokoyama Y Sunami A Hida A. The impact of menstrual cycle phases on cardiac autonomic nervous system activity: an observational study considering lifestyle (diet, physical activity, and sleep) among female college students. J Nutr Sci Vitaminol (Tokyo) (2017) 63:249–55. 10.3177/jnsv.63.249 28978872

[49] Millet GP Roels B Schmitt L Woorons X Richalet JP. Combining hypoxic methods for peak performance. Sports Med. (2010) 40:1–25. 10.2165/11317920-000000000-00000 20020784

[50] Yamamoto Y Hughson RL. Coarse-graining spectral analysis: new method for studying heart rate variability. J Appl Physiol. (1991) 71:1143–50. 10.1152/jappl.1991.71.3.1143 1757311

[51] Howley ET Bassett DRJ Welch HG. Criteria for maximal oxygen uptake: review and commentary. Med Sci Sports Exerc. (1995) 27:1292–301. 8531628

[52] Midgley AW McNaughton LR Polman R Marchant D. Criteria for determination of maximal oxygen uptake: a brief critique and recommendations for future research. Sport Med. (2007) 37:1019–28. 10.2165/00007256-200737120-00002 18027991

[53] Millet GP Candau R Fattori P Bignet F Varray A. VO2 responses to different intermittent runs at velocity associated with VO_2_max. Can J Appl Physiol. (2003) 28:410–23. 10.1139/h03-030 12955868

[54] Fritz CO Morris PE Richler JJ. Effect size estimates: current use, calculations, and interpretation. J Exp Psychol Gen. (2012) 141:2–18. 10.1037/a0024338 21823805

[55] Cohen J Statistical Power Analysis for the Behavioral Sciences. 2nd ed. Hillsdale, NJ: Lawrence Erlbaum Associates (1988). p. 1–567.

[56] Faul F Erdfelder E Lang A-G Buchner A. G^*^Power 3: a flexible statistical power analysis program for the social, behavioral, and biomedical sciences. Behav Res Methods (2007) 39:175–91. 10.3758/BF03193146 17695343

[57] Daimon M Watanabe H Abe Y Hirata K Hozumi T Ishii K. Gender differences in age-related changes in left and right ventricular geometries and functions. Echocardiography of a healthy subject group. Circ J. (2011) 75:2840–6. 10.1253/circj.CJ-11-0364 21946355

[58] Okura H Takada Y Yamabe A Kubo T Asawa K Ozaki T. Age- and gender-specific changes in the left ventricular relaxation: a doppler echocardiographic study in healthy individuals. Circ Cardiovasc Imaging (2009) 2:41–6. 10.1161/CIRCIMAGING.108.809087 19808563

[59] Åstrand P-O Rodahl K Dahl HA Strømme SB Textbook of Work Physiology: Physiological Bases of Exercise. 4th ed New York, NY: McGraw Hill (2003). 1–649 p.

[60] Mujika I Endurance Training—Science and Practice. 1st ed. Vitoria-Gasteiz: Iñigo Mujika (2012). 1–328 p.

[61] Esco MR Williford HN Flatt AA Freeborn TJ Nakamura FY. Ultra-shortened time-domain HRV parameters at rest and following exercise in athletes: an alternative to frequency computation of sympathovagal balance. Eur J Appl Physiol. (2018) 118:175–84. 10.1007/s00421-017-3759-x 29128939

[62] Wang HM Huang SC SDNN/RMSSD as a surrogate for LF/HF: a revised investigation. Model Simul Eng. (2012) 2012:931943 10.1155/2012/931943

[63] Komesaroff PA Esler MD Sudhir K. Estrogen supplementation attenuates glucocorticoid and catecholamine responses to mental stress in perimenopausal women. J Clin Endocrinol Metab. (1999) 84:606–10. 10.1210/jc.84.2.606 10022424

[64] Huikuri HV Pikkujämsä SM Airaksinen KE Ikäheimo MJ Rantala AO Kauma H. Sex-related differences in autonomic modulation of heart rate in middle-aged subjects. Circulation (1996) 94:122–5. 10.1161/01.CIR.94.2.122 8674168

[65] Jones PP Davy KP Seals DR. Influence of age on the sympathetic neural adjustments to alterations in systemic oxygen levels in humans. Am J Physiol. (1997) 273 (2 Pt 2):R690–5. 9277556 10.1152/ajpregu.1997.273.2.R690

[66] Roche F Reynaud C Garet M Pichot V Costes F Barthélémy J-C. Cardiac baroreflex control in humans during and immediately after brief exposure to simulated high altitude. Clin Physiol Funct Imaging (2002) 22:301–6. 10.1046/j.1475-097X.2002.00434.x 12487001

[67] Halliwill JR Minson CT. Effect of hypoxia on arterial baroreflex control of heart rate and muscle sympathetic nerve activity in humans. J Appl Physiol. (2002) 93:857–64. 10.1152/japplphysiol.01103.2001 12183478

[68] Ohyabu Y Honda Y. Exercise and ventilatory chemosensitivities. Ann Physiol Anthropol. (1990) 9:117–21. 10.2114/ahs1983.9.117 2205207

[69] Harms CA McClaran SR Nickele GA Pegelow DF Nelson WB Dempsey JA. Exercise-induced arterial hypoxaemia in healthy young women. J Physiol. (1998) 507 (Pt 2):619–28. 9518719 10.1111/j.1469-7793.1998.619bt.x PMC2230801

[70] Guenette JA Diep TT Koehle MS Foster GE Richards JC Sheel AW. Acute hypoxic ventilatory response and exercise-induced arterial hypoxemia in men and women. Respir Physiol Neurobiol. (2004) 143:37–48. 10.1016/j.resp.2004.07.004 15477171

[71] Harms CA. Does gender affect pulmonary function and exercise capacity? Respir Physiol Neurobiol. (2006) 151:124–31. 10.1016/j.resp.2005.10.010 16406740

[72] Wilmore JH Costill DL Physiology of Sport and Exercise. 3rd ed. Champaign, IL: Human Kinetics (2004) 1–726 p.

[73] Moore LG Cymerman A Huang SY McCullough RE McCullough RG Rock PB Propranolol blocks metabolic rate increase but not ventilatory acclimatization to 4300 m. Respir Physiol. (1987) 70:195–204. 10.1016/0034-5687(87)90050-8 3671899

[74] Bayliss DA Millhorn DE. Central neural mechanisms of progesterone action: application to the respiratory system. J Appl Physiol. (1992) 73:393–404. 10.1152/jappl.1992.73.2.393 1399957

[75] Tatsumi K Pickett CK Jacoby CR Weil JV Moore LG. Role of endogenous female hormones in hypoxic chemosensitivity. J Appl Physiol. (1997) 83:1706–10. 10.1152/jappl.1997.83.5.1706 9375342

[76] Lusina S-JC Kennedy PM Inglis JT McKenzie DC Ayas NT Sheel AW. Long-term intermittent hypoxia increases sympathetic activity and chemosensitivity during acute hypoxia in humans. J Physiol. (2006) 575 (Pt 3):961–70. 10.1113/jphysiol.2006.114660 16809359 PMC1995690

[77] Schwartz J Katz SA Fegley RW Tockman MS. Sex and race differences in the development of lung function. Am Rev Respir Dis. (1988) 138:1415–21. 10.1164/ajrccm/138.6.1415 3202496

[78] Mead J. Dysanapsis in normal lungs assessed by the relationship between maximal flow, static recoil, and vital capacity. Am Rev Respir Dis. (1980) 121:339–42. 7362140 10.1164/arrd.1980.121.2.339

[79] Levental S Picard E Mimouni F Joseph L Samuel TY Bromiker R. Sex-linked difference in blood oxygen saturation. Clin Respir J. (2018) 12:1900–4. 10.1111/crj.12753 29227023

[80] Humpeler E Vogel S Schobersberger W Mairbäurl H. Red cell oxygen transport in man in relation to gender and age. Mech Ageing Dev. (1989) 47:229–39. 10.1016/0047-6374(89)90035-3 2716369

